# *Agrobacterium tumefaciens*-mediated transformation and expression of GFP in *Ascochyta lentis* to characterize ascochyta blight disease progression in lentil

**DOI:** 10.1371/journal.pone.0223419

**Published:** 2019-10-24

**Authors:** Bernadette M. Henares, Johannes W. Debler, Lina M. Farfan-Caceres, Christina R. Grime, Robert C. Lee

**Affiliations:** Centre for Crop and Disease Management, School of Molecular and Life Sciences, Curtin University, Bentley, WA, Australia; University of Southern Queensland, AUSTRALIA

## Abstract

The plant immune system is made up of a complex response network that involves several lines of defense to fight invading pathogens. Fungal plant pathogens on the other hand, have evolved a range of ways to infect their host. The interaction between *Ascochyta lentis* and two lentil genotypes was explored to investigate the progression of ascochyta blight (AB) in lentils. In this study, we developed an *Agrobacterium tumefaciens*-mediated transformation system for *A*. *lentis* by constructing a new binary vector, pATMT-GpdGFP, for the constitutive expression of green fluorescent protein (EGFP). Green fluorescence was used as a highly efficient vital marker to study the developmental changes in *A*. *lentis* during AB disease progression on the susceptible and resistant lentil accessions, ILL6002 and ILL7537, respectively. The initial infection stages were similar in both the resistant and susceptible accessions where *A*. *lentis* uses infection structures such as germ tubes and appressoria to gain entry into the host while the host uses defense mechanisms to prevent pathogen entry. Penetration was observed at the junctions between neighbouring epidermal cells and occasionally, through the stomata. The pathogen attempted to penetrate and colonize ILL7537, but further fungal advancement appeared to be halted, and *A*. *lentis* did not enter the mesophyll. Successful entry and colonization of ILL6002 coincided with structural changes in *A*. *lentis* and the onset of necrotic lesions 5–7 days post inoculation. Once inside the leaf, *A*. *lentis* continued to grow, colonizing all parts of the leaf followed by plant cell collapse. Pycnidia-bearing spores appeared 14 days post inoculation, which marks the completion of the infection cycle. The use of fluorescent proteins in plant pathogenic fungi together with confocal laser scanning microscopy, provide a valuable tool to study the intracellular dynamics, colonization strategy and infection mechanisms during plant-pathogen interaction.

## Introduction

Lentil (*Lens culinaris* Medik.) is one of the earliest domesticated crops and is sometimes referred to as one of the ancient grains in modern times [1]. This high-value crop is an excellent source of high quality plant protein for human nutrition. Average annual global production of lentil was reported to be 5.07 million metric tonnes from 2010 to 2017. Production is concentrated in Canada, India, Turkey, United States of America and Australia, which contributed to a combined total of more than 75% of the world’s lentil production [2]. The Australian lentil industry produces an average of 292,000 tonnes annually and production is centred in the Southern temperate cropping zones of South Australia and Victoria [3]. Lentil ascochyta blight (AB) is a major threat to lentil production worldwide and is caused by the necrotrophic ascomycete fungal pathogen *Ascochyta lentis*, a member of the *Didymellaceae* [4]. *A*. *lentis* produces necrotic lesions on the above ground parts of the plant resulting in stem damage, loss of leaves and pod discoloration [5]. In severely infected crops, necrotic lesions on leaves and pods lead to reduced yield and poor seed quality [6]. Pathogen populations persist in lentil cropping areas by growth on plant residues and stubble at the start of the next growing season and adaptation and distribution of *A*. *lentis* are facilitated through sexual recombination between compatible mating types and the production of windborne ascospores. Average annual yield and grain quality losses in Australia amount to A$0.9 M and control costs add a further A$15.3 M penalty to the Australian lentil industry [7].

As is the case for ascochyta blight of other pulse crops, *A*. *lentis* is difficult to control. Management strategies include cultural practices, fungicide treatment of seed and by foliar application [8], and use of resistant cultivars [9]. Plant breeding for AB resistance has provided some degree of protection [9,10], but the durability of plant resistance has often been challenged by the ability of pathogen populations to evolve new virulence forms or levels of aggressiveness [11]. Adaptation of pathogens at the population level often leads to novel pathotypes that can result in breakdown of resistance genes. Changes in aggressiveness and shifts in cultivar specificity or virulence are a constant challenge for growers and breeders [12]. Thus, many studies have aimed to investigate the interaction of *A*. *lentis* with different lentil genotypes [13–16]. Studies on the early infection process on detached leaf samples have revealed that resistant lentil genotypes recognised and reacted faster to pathogen infection [13–16]. Recognition of the pathogen triggers a cascade of cellular and molecular responses. Defense signalling pathway and key defense response genes have been identified through transcriptome profiling of lentil genotypes with non-allelic AB resistance genes to *A*. *lentis* [17,18]. Genes associated with pathogen recognition as well as genes that comprise salicylic acid, abscisic acid and jasmonic acid transduction pathways were differentially expressed in a genotype-dependent manner [17]. On the pathogen side, *A*. *lentis* isolates exhibited delayed and reduced formation of infection structures in resistant varieties compared to susceptible varieties. Histopathological studies using differential staining and monitoring of the production of reactive oxygen species (ROS) have been widely used for studying plant-pathogen interactions including *A*. *lentis* in lentil [14,19]. Diaminobenzidine (DAB) staining is effective for detection of host responses where ROS are produced by host cells and Trypan Blue stain enables specific staining of mycelium and other fungal structures on plant surfaces. However, staining techniques are most suited to observation of fungal development on host surfaces that pertain to the early colonization phase of infection. The detection of fungal invasion of epidermal and mesophyll layers that characterize late stages of the infection cycle for fungal pathogens is limited by the inefficient staining of fungal structures inside intact plant tissues.

The use of fluorescent reporter proteins, such as green fluorescent protein (EGFP), is a powerful, non-destructive strategy for the study of fungal pathogens and the interaction with their respective hosts. Fluorescence microscopy, and the more sensitive confocal laser scanning microscopy (CLSM), enable the observation of fluorescent fungal structures, both on the surface and inside infected tissues. Various plant pathogenic fungi have been transformed with constructs for the expression of GFP, including *Leptosphaeria maculans* [20] *Sclerotinia sclerotiorum* [21], *Parastagonospora nodorum* [22] and *Ascochyta rabiei* [23]. This approach has allowed all infection stages of early colonization, intracellular growth and sporulation to be observed *in planta* and analysed non-invasively in living cells and tissues. The combination of fluorescent tagging and advanced microscopy offers new possibilities for studying pathogen-host interactions.

Information on the development of transformation methods for members of the *Didymellaceae* family is very limited. Previous studies have reported the transformation of the hygromcyin resistance gene and the *gfp* gene into *A*. *rabiei* using *Agrobacterium tumefaciens*-mediated transformation (ATMT) [23,24], but none so far has been reported for *A*. *lentis*. Here, we describe the first application of ATMT in *A*. *lentis* and report the use of an EGFP-expressing *A*. *lentis* strain to characterize the interaction of *A*. *lentis* with lentil host genotypes that differ in susceptibility and pathogen responses. Furthermore, this study demonstrates that we can monitor AB disease progression in lentils for the full infection cycle using CLSM.

## Materials and methods

### Materials, microbial strains and plasmid vector

*Ascochyta lentis* Kewell strain (named *Al*Kewell hereafter) [12,14] was used as a recipient strain for transformation and was grown in half strength Potato Dextrose Agar (½ PDA) (BD Difco, Sparks, MD, USA). Sambasivam et al. (2017) [14] described responses of lentil accessions ILL6002 and ILL7537 to *Al*Kewell as moderately resistant and resistant, respectively. In our bioassay conditions, at 14 days after inoculation, *Al*Kewell produces more distinct disease responses in these lentil accessions with 30% leaf area damage and no disease, respectively. Maintenance and spore preparation of *A*. *lentis* isolates were performed as described by Davidson et al. [12]. The AGL1 strain of *A*. *tumefaciens* was used for ATMT. The *Escherichia coli* strain One Shot OmniMAX 2^TM^ T1 (OM2) (Invitrogen, Carlsbad, USA) was used for cloning and construction of plasmids. Plasmids were maintained in OM2. All reagents were from Sigma-Aldrich (St. Louis, MO, USA), unless otherwise indicated.

### Resistance level of *Ascochyta lentis* Kewell to hygromycin B

The *Al*Kewell strain was grown in ½ PDA plates supplemented with hygromycin B (Invitrogen, Carlsbad, USA) at concentrations: 0, 50, 100, 150 and 200 ng.μL^-1^ and incubated at 25°C in the dark. Hyphal growth was monitored daily until the colony covered two-thirds of the plate. Growth was totally inhibited at 50 ng.μL^-1^ and this concentration was used for selection of putative transformant colonies.

### Plant material

Lentil accessions that differ in the disease response to *Al*Kewell, ILL6002 and ILL7537 were used in this study. Lentil accessions ILL6002 and ILL7537 were susceptible and resistant controls, respectively. Lentil varieties were obtained from Seednet (Horsham, Australia) and from Pulse Breeding Australia (Horsham, Australia). Three lentil seeds were sown 1 cm deep in 10 cm pots filled with soil (UWA mix, Richgro, Jandakot, WA, Australia) (five replicates per host genotype-isolate combination). Plants were grown for two weeks in a growth chamber maintained at 18 ± 2°C with a 12/12-hour photoperiod. LED lights were Lumigrow Lumibar 200W (LumiGrow, Emeryville, CA, USA) and S-tech LED 108D18-12 18W tubes (S-tech, Canning Vale, WA, Australia).

### Plasmid construction

The binary vectors used for ATMT were constructed using parts of the two plasmids, pEAQ-HT-DEST1 [25] and pGpdGFP [20]. A 4.8 Kb fragment containing the ColEI, OriV, a kanamycin resistance gene (*nptIII*) and *trfA*, left and right border sequences were PCR-amplified from pEAQ-HT-DEST1 (kindly provided by G. Lomonossoff and F. Sainsbury, John Innes Centre, UK) using the primers JD278 and JD287 (S1 Table). The 4 Kb fragment containing the *trp*C promoter, hygromycin resistance gene (*hph*), *gpd*A promoter, *egfp* gene and *trp*C terminator were amplified from pGpdGFP [20] (obtained from R. Oliver, Curtin University) using primers JD292 and JD293 (S1 Table). The PCR of both fragments was carried out in 20 μL reactions containing 1 μL Phusion U Hot Start DNA polymerase (2 U/μL) (Thermo Fisher Scientific), 4 μL 5x HF buffer and 0.2 μM of each primer. Amplification/cycling conditions were: denaturation for 30 s at 98°C, 35 cycles of 10 s at 98°C, 20 s at 55°C and 3 min at 72°C, final elongation for 10 min at 72°C. The PCR products were digested with the USER^TM^ enzyme mix (NEB, Ipswich, MA, USA) according to manufacturer’s instructions to remove deoxyuracil residues. Briefly, PCR products of equal concentration were mixed and 8 μL of the mixture were combined with 1 μL of 10x Cutsmart buffer and 1 U of USER enzyme (NEB, 1000 U/mL). The reaction mixture was incubated for 20 mins at 37°C followed by incubation at 25°C for 20 minutes. The resulting vector, pATMT-GpdGFP (S1 Fig), was transformed into competent OM2 *E*. *coli* cells and plated onto LB + kanamycin plates (30 ng. μL ^-1^). Putative transformants were screened using PCR to select for constructs having the correct arrangement of components. Verification of the final construct was determined by Sanger sequencing across the junctions between fragments. The binary vector, pATMT-GpdGFP, was produced in OM2 *E*. *coli* and *A*. *tumefaciens* AGL1 was transformed by electroporation of competent AGL1 cells.

### *Agrobacterium tumefaciens*-mediated transformation (ATMT) of *Ascochyta lentis*

AGL1 harboring pATMT-GpdGFP vector was grown in LB medium containing 50 μg.mL^-1^ rifampicin (Astral Scientific, Gymea, NSW, Australia) and 30 μg.mL^-1^ kanamycin (Astral Scientific, Gymea, NSW, Australia), at 28°C and 250 rpm shaking, until approx. OD_600nm_ 0.5. The culture was washed twice with induction medium (IM: Minimal media salts, 40 mM 2-(N-morpholino)ethanesulfonic acid (MES) (pH5.3), 10 mM glucose and 0.5% [w/v] glycerol. MM salts contain 2.05 g K_2_HPO_4_, 1.45 g KH_2_PO_4_, 0.15 g NaCl, 0.5 g MgSO_4_·7H_2_O, 0.1 g CaCl_2_·6H_2_O, 0.0025 g FeSO_4_·7H_2_O and 0.5 g (NH_4_)_2_SO_4_ per litre) and resuspended at approx. OD_600nm_ 0.5 in the same medium containing 200 μM acetosyringone [26]. Spores of *Al*Kewell from a one week-old ½ PDA plate were washed twice with water, filtered through sterile cotton wool inside a five ml plastic syringe, and resuspended in water to a final concentration of approx. 2 x 10^7^ spores.mL^-1^. Equal volumes of *Al*Kewell spore suspension and the AGL1 transformant carrying pATMT-GpdGFP were co-cultivated on a filter membrane (cellulose nitrate 0.45 μm, Whatman^TM^, Buckinghamshire, UK) on IM plates [26] containing 200 μM acetosyringone for two days in the dark at 25°C. Following co-incubation, membranes were transferred to ½ PDA plates containing 50 ng.μL^-1^ hygromycin B, 50 ng.μL^-1^ cefotaxime (Astral Scientific, Gymea, NSW, Australia) and 30 ng.μL^-1^ streptomycin (Astral Scientific, Gymea, NSW, Australia) and incubated at 25°C in the dark until colonies appeared. Colonies that appeared 7–10 days after incubation were transferred to fresh selection plates containing antibiotics. The putative transformants were maintained on ½ PDA plates containing antibiotics. The stability of *A*. *lentis* transformants resistant to hygromycin B was tested by sub-culturing five times on ½ PDA plates without hygromycin B, after which transformants were transferred to PDA plates containing hygromycin B (50 ng.μL^-1^).

Putative *A*. *lentis* transformants were screened for positive EGFP expression by monitoring fluorescence using a Nikon A1+ Confocal cell imaging system (Nikon, Tokyo, Japan) using filters at 488 nm excitation and 525/50 nm emission. EGFP-fluorescent transformants were stored at -80°C as a spore suspension in 15% glycerol until further analysis. All experiments were initiated from stored cultures.

### Molecular characterization of *Al*Kewell82-GFP

From multiple colonies resistant to antibiotics, the stable transformant, *Al*Kewell82-GFP, was grown in yeast extract dextrose liquid media with constant shaking at 180 rpm for 72 hours at 22°C in the dark. Mycelium samples were frozen with liquid nitrogen and ground to a fine powder. Approximately 500 mg of powdered mycelium was used for DNA extraction using a modified cetyltrimethylammonium bromide (CTAB) method [27]. Briefly, DNA samples were dissolved in 10 mM Tris-HCl, pH 8 and purified with AMPure XP magnetic beads (Agencourt, Beckman Coulter, Brea, CA, USA) following manufacturer recommendations. DNA concentration was determined using a Qubit® 2.0 fluorometer (Invitrogen, Carlsbad, USA) with the dsDNA Broad range assay kit (Thermo Fisher Scientific) and NanoDrop Spectrophotometer (Thermo Fisher Scientific), and DNA quality was assessed by agarose gel electrophoresis (1%).

To validate and to characterize the integration site of the GFP expression cassette into the genome of *Al*Kewell82-GFP, we carried out Illumina sequencing on total DNA from the *Al*Kewell82-GFP strain. Sequencing was performed by Novogene on an Illumina HiSeq (Illumina, San Diego, CA, USA). Raw 150 bp paired end reads were trimmed with Trimmomatic version 0.38 [28] and *de novo* assembled with SPAdes version 3.13.0 [29]. Reads were mapped back against the *Al*Kewell82-GFP *de novo* assembly using HISAT2 version 2.1.0 [30]. The contig with the GFP cassette insertion site was determined using BLASTn with the *egfp* nucleotide sequence against the *de novo Al*Kewell82-GFP assembly. The insertion site was confirmed by PCR with primer pairs JD425 and JD381 and JD427 and JD321 (S1 Table).

### Evaluation of growth and sporulation of *Al*Kewell82-GFP

The stably fluorescing transformant *Al*Kewell82-GFP was chosen for further studies with respect to virulence, growth rate and sporulation. Characterization of the EGFP-expressing *A*. *lentis* transformant was undertaken using spore suspension harvested from a one-week old plate as described above. The spore concentration was determined using a haemocytometer and adjusted to approximately 1 x 10^6^ spores.mL^-1^. A sterile circular (0.5 cm diameter) paper filter disk (Whatman^TM^, Buckinghamshire, UK) was dipped in the spore suspension and placed in the middle of a ½ PDA plate without hygromycin B and allowed to grow in an incubator with 12/12-hour near ultra-violet light/dark cycles at room temperature. Mycelial growth was monitored regularly until the colony covered half of the plate. Measurements were reported as the diameter of the mycelial area. Spores and mycelia from the plate were collected and mounted on a glass slide for microscopic examination to assess EGFP expression *in vitro* using CLSM.

Virulence of *Al*Kewell82-GFP was assessed on the susceptible lentil accession ILL6002 and compared to untransformed *Al*Kewell. Whole plant infection assay was performed as described by Davidson et al. [12] with modifications. Briefly, spores were prepared as described above. Two-week old plants were spray-inoculated until run-off, making sure of an even distribution of inoculum by spraying plants all together as one group. Inoculated plants were kept in a chamber with misting to ensure the chamber environment was under high humidity. The whole chamber was misted for five seconds every two hours for nine days. The room temperature was maintained at 18°C with a 12/12-hour photoperiod. Macroscopic lesions appeared 6–8 days post infection (DPI). Disease symptoms were evaluated at 14 DPI and scores were reported as % leaf area damage (LAD). Three independent experiments were carried out for each lentil genotype.

### Confocal laser scanning microscopy of infected lentil accessions

Development of fungal structures and disease progression of *Al*Kewell82-GFP were monitored over a period of 14 days. Samples were collected at 1, 3, 5, 7, 9, and 14 DPI. A minimum of three leaves were collected per lentil genotype for each time point. At early time points up until day 5, there were no observable symptoms on either accession. However, at later time points symptoms were observed in accordance with the natural progression of the disease as recorded in which approximated 30% leaf area damage as evidenced by tissue necrosis at 14 DPI. The whole leaf was mounted on a microscope slide with 20% glycerol and covered with a cover slip. Fluorescence images were acquired using an inverted Nikon A1+ confocal microscope with NIS-Elements confocal software (Nikon Instruments, Tokyo, Japan). Images were collected at digital scan resolution (pixel dwell = with 1024 resolution using a 20x Plan Apo dry objective). Sequential laser scanning was performed using four lasers: 405 nm (450/50 filter), 488 nm (525/50 filter), 561 nm (595/50 filter) and 640 nm (700/75 filter) to view nuclei, GFP, and chloroplast and cell wall autofluorescence. The top and bottom positions of the field of view for each sample were set using the NIS-Elements microscope control software and multiple images were taken in Z-series. Three-dimensional representations and 2D Z-projections were produced using Fiji ImageJ software, version 1.51w [31].

### Propidium iodide staining

Leaf tissues from infected lentil accession ILL6002 was stained with propidium iodide (PI) at a concentration of 10 μg.mL^-1^ as described by Jones et al. [32]. Observation of PI-stained leaves was carried out using Nikon A1+ confocal microscope with 561 nm excitation and 595/50 nm emission. Images were processed using ImageJ, version 1.51w [31].

### Histochemical detection of hydrogen peroxide (H_2_O_2_)

The production of H_2_O_2_ by ILL6002 in response to *A*. *lentis* WT and *Al*Kewell82-GFP was detected using the 3,3-diaminobenzidine (DAB) uptake method as described by Sambasivam et al. [14]. Briefly, a minimum of three infected lentil leaf tissue samples were collected at one DPI for each isolate and incubated in DAB solution (1 mg.mL^-1^) for approximately eight hours in the dark at room temperature. Tissues were then cleared by immersion in ethanol:glacial acetic acid solution (1:2 V/V) for at least 24 hours to clear the chlorophyll. Fungal structures were then stained with Trypan Blue (0.05%; Sigma-Aldrich) for 10 mins at room temperature [14]. Stained leaf samples were mounted on a microscope slide with 20% glycerol and covered with cover slip. Production of ROS was detected as a reddish-brown coloration while fungal structures appeared blue. Samples were examined with Olympus BX51 microscope (Olympus, Tokyo, Japan) fitted with a digital camera. Images were captured using the Olympus DP controller image capture software.

## Results

### Construction of the pATMT-GpdGFP binary vector and transformation of *Ascochyta lentis*

For the development of a method for genetic transformation of *A*. *lentis* using A*grobacterium tumefaciens*-mediated transformation (ATMT), we constructed a new binary vector, pATMT-GpdGFP, using parts of the two plasmids, pEAQ-HT-DEST1 [25] and pGpdGFP [20]. The regions essential for replication in *E*. *coli* were amplified from pEAQ-HT-DEST1 which included the ColEI and OriV replication origins, a kanamycin resistance gene (*nptIII*), the plasmid replication initiator protein trfA, as well as the left and right border sequences necessary for *Agrobacterium*-mediated integration into the target fungal genome [25]. The transgene components containing the hygromycin resistance gene (*hph*) and e*gfp* gene expression cassettes were amplified from pGpdGFP. Both fragments were amplified using primers containing short overlapping tails that allow for targeted and directional fusion of fragments using USER^TM^ cloning [33]. PCR fragments were digested with USER^TM^ enzyme mix to remove deoxyuracil residues incorporated into the primers. Subsequent dissociation of the remnant short single-stranded DNA upstream from the dU site produced single-stranded ends for annealing to adjacent PCR fragments in the final construct. The resulting T-DNA was flanked by left and right borders and contained the hygromycin selectable marker with the hygromycin B phosphotransferase resistance gene (*hph*) driven by the *Aspergillus nidulans* tryptophan C promoter (P*trp*C), and the reporter gene *egfp* under the control of the glyceraldehyde-3-phosphate dehydrogenase promoter (P*gpdA*) (S1 Fig) The plasmid sequence of pATMT-GpdGFP can be found in the Supporting Information (S1 and S2 Files).

For the method development of ATMT in *A*. *lentis*, we first determined the sensitivity of the fungal isolate to different concentrations of hygromycin B (50, 100, 150 and 200 ng.μL^-1^). Fungal growth was not observed at 50 ng.μL^-1^ hygromycin B and this concentration was therefore used to select putative transformants in subsequent experiments. Transformation was achieved by combining *A*. *tumefaciens* AGL1 carrying pATMT-GpdGFP with *Al*Kewell spores, and co-culturing on nitrocellulose membranes on solid media containing acetosyringone without antibiotic to enable recovery of fungal transformants. The membrane containing the co-cultures was then transferred onto a selection plate containing hygromycin B, cefotaxime and streptomycin. Colonies that appeared 7–10 days after co-incubation were subcultured onto fresh selection plates to ensure that the recovered transformants were not false positives. Colonies resistant to hygromycin B were selected for further analysis, including PCR detection of both the hygromycin B selectable marker and *egfp* genes, and the ability to express EGFP both *in vitro* and *in planta*, tested using fluorescence microscopy. Several single spore isolates showed fluorescence with varying degrees of intensity. However, only stable *Al*Kewell-GFP transformant that maintained a high level of EGFP expression after repetitive subculture on a non-selective medium was chosen for further studies.

The *A*. *lentis* transformant, *Al*Kewell82-GFP was chosen for spectral confocal microscopy and plant-pathogen interaction studies because it showed strong and uniform green fluorescence. Expression of EGFP was clearly evident at the different stages of fungal development, including vegetative hyphae and pycnidiospores, when grown on PDA containing hygromycin B (S2A Fig). Moreover, we monitored EGFP expression of *Al*Kewell82-GFP *in planta*, where we observed strong green fluorescence in mycelia at three DPI (S2B Fig) and pycnidiospores that formed at 14 DPI on the susceptible lentil accession, ILL6002 (S2C Fig).

### Molecular analysis of *egfp* gene integration into the fungal genome

**Fig 1:**
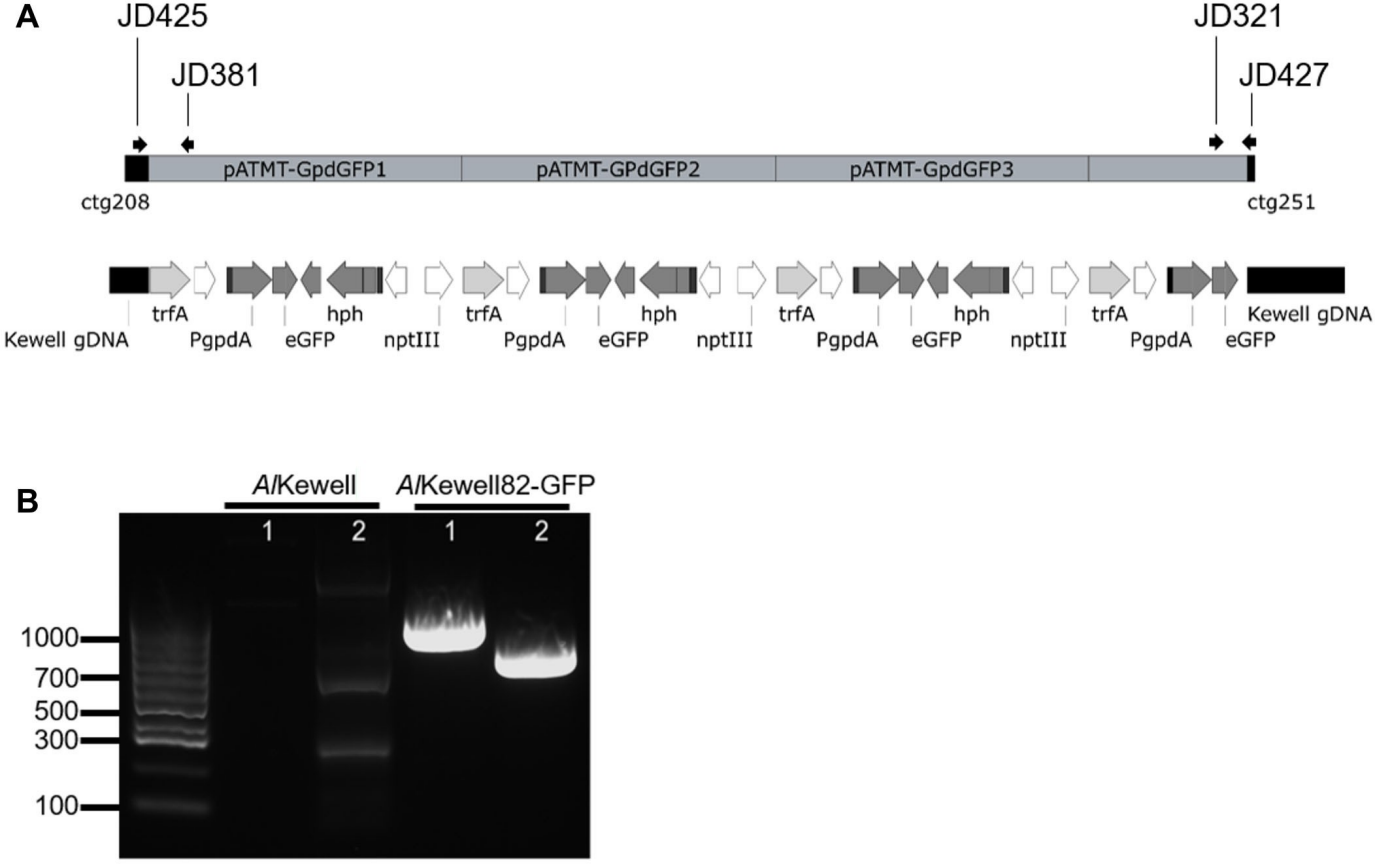
(A) Schematic diagram of tandem T-DNA sequences at the insertion site in *Al*Kewell82-GFP prepared by SnapGene Viewer (GSL Biotech; available at snapgene.com) (B) PCR detection of the T-DNA and flanking host sequences using primers JD425 and JD381 (917 bp) and JD321 and JD427 (707 bp) for bands 1 and 2, respectively, and indicated in Fig 1A.

The insertion of T-DNA into the genome in *Al*Kewell82-GFP was verified by Illumina whole genome sequencing. A total of 4.43Gbp of data was *de novo* assembled resulting in a genome assembly of 41.4 Mbp, with an average read coverage of 107x (S2 Table). Using BLASTn with the coding sequence for the *egfp* gene against the *de novo Al*Kewell82-GFP assembly identified the contig with the T-DNA insertion, which revealed the integration of not only the T-DNA but also the vector backbone in a single site in the genome. The insertion site was found to be in the middle of a gene that is annotated by InterProScan as a glycosyltransferase. The glycosyltransferase gene in *Al*Kewell contains six exons where the first three exons were annotated as a glycosyltransferase domain and the last three exons encode a domain of unknown function (DUF). In *Al*Kewell82-GFP, the binary plasmid containing the T-DNA interrupted this gene where the third exon (ctg2018) was potentially fused to *trfA* as predicted by Genemark, while the DUF (ctg251) was annotated as a separate gene. The orientation of the binary plasmid insertion is depicted in **Fig 1A**. From the number of reads mapping to and around the insertion site, we suspect that three copies of the complete plasmid and four full copies of *egfp* and the *gpdA* promoter driving its expression have been integrated into the genome. The average sequencing depth for *A*. *lentis* genomic sequence for *Al*Kewell82-GFP was 107x, and for the vector backbone and for the *egfp* expression cassette sequences, 300x and 400x, respectively (S3 Fig). We validated the integration of the T-DNA into the genome of *Al*Kewell82-GFP by amplifying the flanking regions of the *A*. *lentis* genome and a region in the plasmid using PCR (**Fig 1B**). The 917 bp and 707 bp bands confirmed the integration of the plasmid from the *trfA* gene into the *Al*Kewell82-GFP genome.

**Fig 2:**
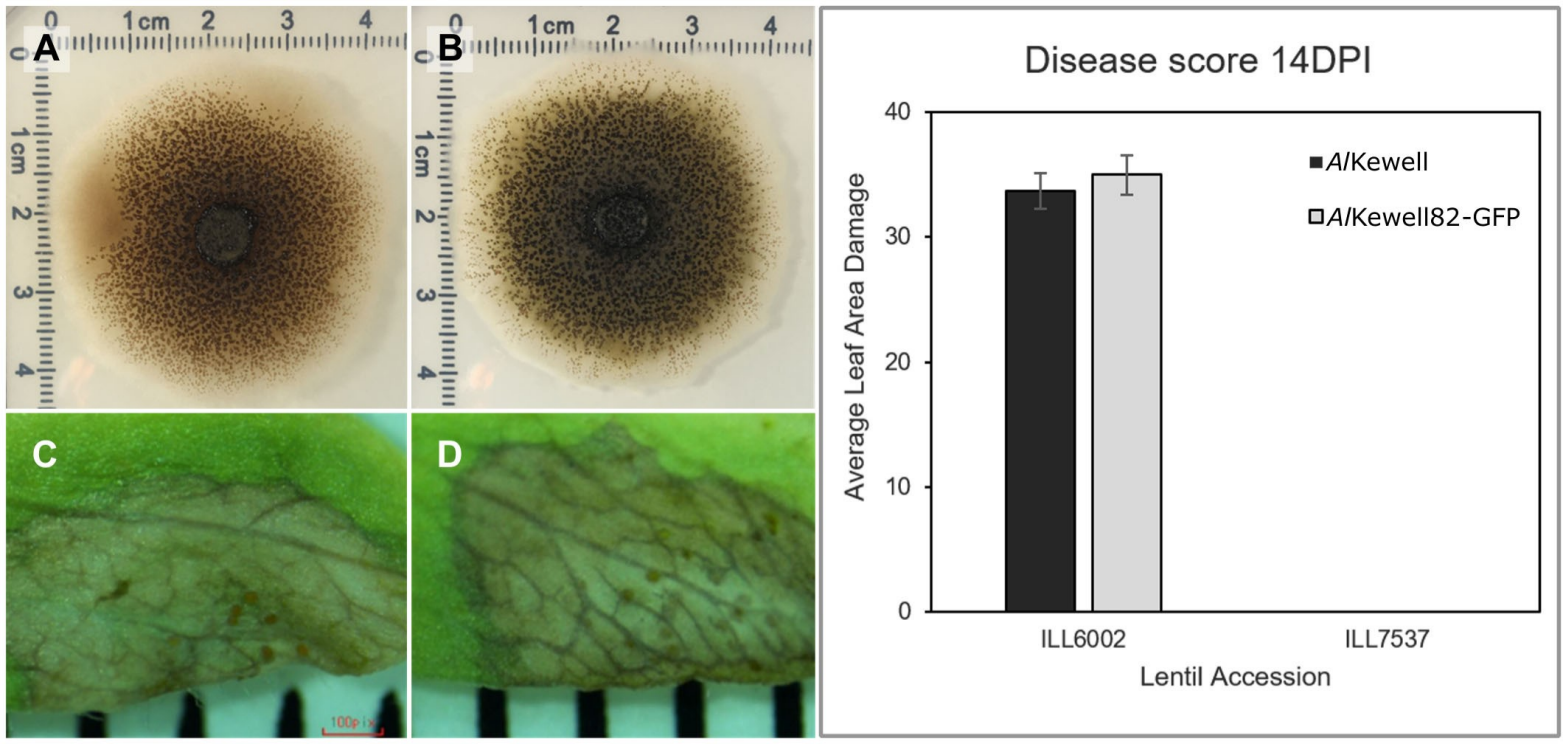
Characterization of *Al*Kewell82-GFP transformant. Radial growth and sporulation of (A) untransformed *Al*Kewell and (B) *Al*Kewell82-GFP. Sporulation of (C) *Al*Kewell and (D) *Al*Kewell82-GFP on ILL6002. (E) Pathogenicity of *Al*Kewell and *Al*Kewell82-GFP on ILL6002 and ILL7537.

To determine the effect of multiple in-tandem insertion and the disruption of the putative glycosyltransferase gene, *Al*Kewell82-GFP was further characterized to ensure that it did not phenotypically vary from the wild-type, *Al*Kewell. Radial mycelial growth and sporulation of *Al*Kewell82-GFP and *Al*Kewell showed no difference when grown on ½ PDA plates after seven days of incubation (**Figs 2A and 2B and** S4). Similarly, the ability of *Al*Kewell82-GFP to cause necrosis and to form pycnidia on ILL6002 14 days post infection was comparable to *Al*Kewell-WT (**Figs 2C and 2D and** S5).

Finally, *Al*Kewell82-GFP virulence on the susceptible ILL6002 and resistant ILL7537 lentil accessions was evaluated. A disease severity of 35% on ILL6002 by *Al*Kewell82-GFP was found to be not significantly different from the untransformed *Al*Kewell at 34%, and both strains were not virulent on ILL7537 (**Fig 2E**) as evidenced by the absence of any observable disease symptoms.

### Fungal development during early stages of host infection

**Fig 3:**
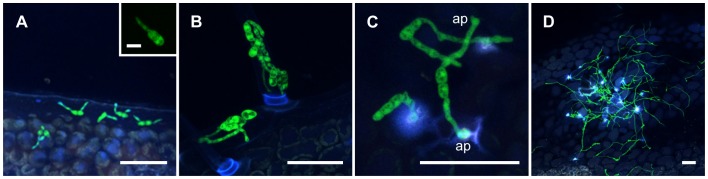
Fungal development during early stages of infection (A) Germination of conidia on the leaf cuticle at one 1 DPI (B) trichomes at 1 DPI (C) Formation of an appressorium (ap) at one DPI; (D) *Al*Kewell82-GFP mycelia after five DPI. Scale bars are 50 μm, inset: scale bar is 10 μm.

We monitored the infection cycle of *Al*Kewell82-GFP under our standard seedling infection assay conditions on the susceptible variety ILL6002. We followed fungal development using confocal microscopy from the time of inoculation until 14 DPI. Conidial germination occurred within 24 hours upon making contact with the leaf surface, consistent with previous studies [13–15]. Analysis of confocal microscope optical sections showed that spores adhered on the cuticle at the leaf surface and occasionally around the trichomes (**Fig 3A and 3B**). Germ tubes emerging from the spores were typically unipolar, but two germ tubes originating from occasional spores were also observed. The germ tube was observed to extend to varying lengths before forming an appressorium (**Fig 3C**). However, not all germ tubes produced an appressorium with some continuing to grow from the apex to form extended hyphae. Within 3–5 days after inoculation, infection hyphae grew long and thin on the host surface and attempts to penetrate the host were observed throughout the early growth stage up to five DPI (**Fig 3D**) without any macroscopic signs of infection or necrosis.

**Fig 4:**
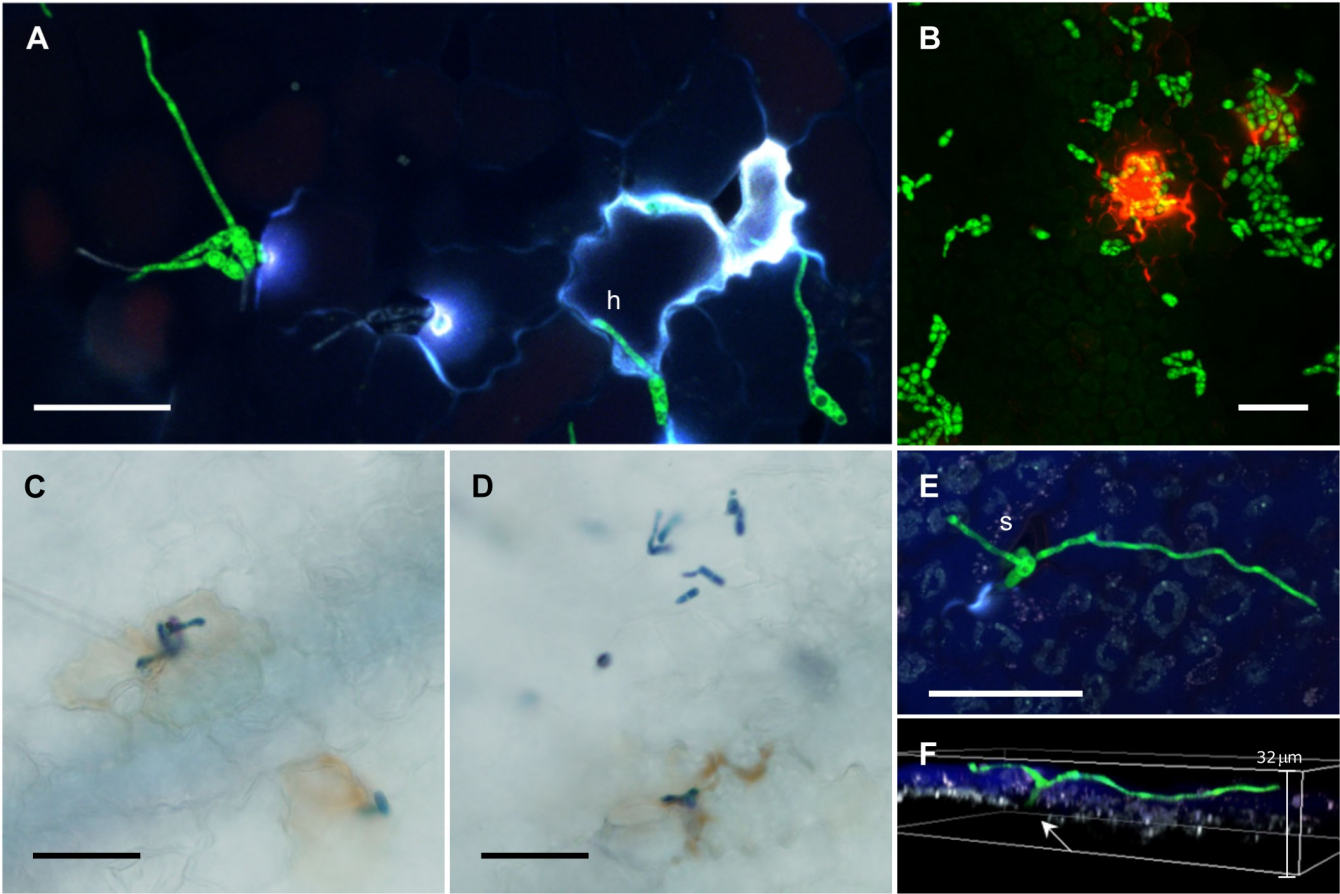
Penetration structures and early colonization events. (A) Unlobed hyphae (h) at one DPI (B) Propidium iodide-stained ILL6002 infected with *Al*Kewell82-GFP. ROS production by ILL6002 in response to (C) *Al*Kewell and (D) *Al*Kewell82-GFP at one DPI (E) Surface growth of *Al*AlKewell82-GFP hyphae and stomatal entry (s). (F) 3D representation of (E) with stomatal cavity indicated (arrow). All scale bars are 50 μm.

Penetration of the host leaf surface was evident as early as 24 hours post inoculation. Penetration structures were identified by the localised swelling of hyphal tips to form appressoria (**Fig 3C**). However, the most commonly observed mode of entry was penetration of the host surface without the formation of an obvious appressorial structure. Alternative penetration structures adopted the form of a simple unlobed structure that was slightly swollen at the tip. These penetration structures were most often found near the groove or at the junction between epidermal cells (**Fig 4A**). The response of ILL6002 to *Al*Kewell82-GFP was indicated by the presence of elevated white autofluorescence signals around the appressoria and the hyphal tips of the green fluorescent fungi. White fluorescence results from the co-localization of multiple signals from all detector channels. The fluorescence signal at the site of penetration indicates the release of phenolic compounds from the host due to disruption of the cell protoplasm [15,22]. We confirmed the localised host response using propidium iodide (PI) that stains dead cells. It was evident from the red fluorescent signal (wavelength range: 595/50 nm), that there are dead plant cells at the site of penetration or possibly damage to the cuticle or epidermal layer that has allowed the propidium iodide stain to penetrate tissues and label cell walls [32]. Taken together, fluorescence signals with or without propidium iodide stain, clearly indicate that *A*. *lentis* mycelia have attempted, and in some cases succeeded in penetrating the host tissue (**Fig 4B**). In addition, formation of reactive oxygen species (ROS), a known host defense response, was detected using DAB stain by light microscopy within 24 hours of infection (**Fig 4C and 4D**).

Fungal entry through the stomatal opening was also observed (**Fig 4E**). The germ tube grew at random without moving directly towards the stomata. Interestingly, Z-projections of confocal optical sections from the leaf surface to beneath the epidermis (approx. 10 μm) revealed that entry through the stomata did not show elevated white autofluorescence around the stomata which suggests that the pathogen was able to enter the host without the plant eliciting/producing phenolic compounds or a cell death response.

### Cellular reaction of susceptible and resistant lentil varieties to *Al*Kewell82-GFP

**Fig 5:**
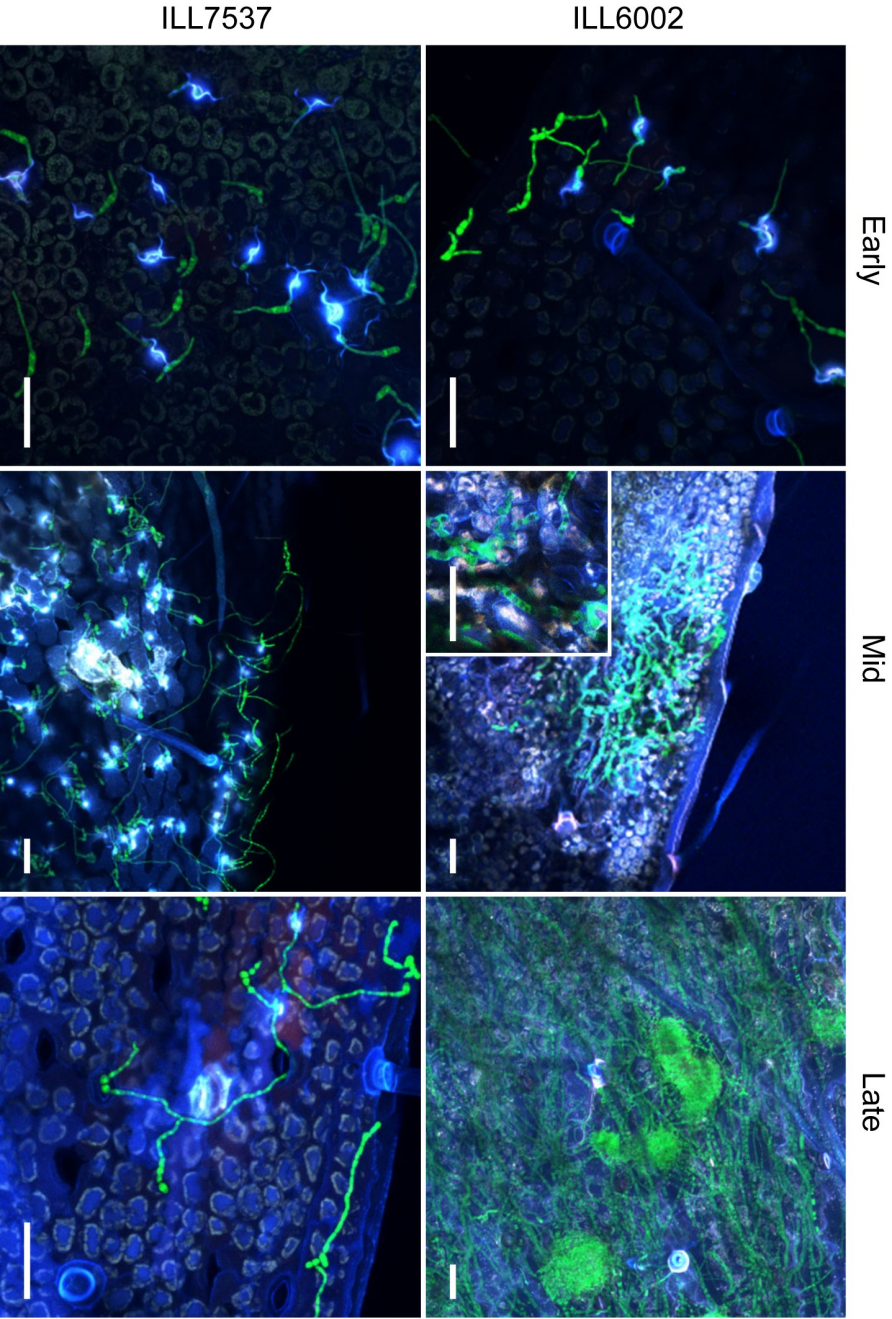
Cellular reaction of susceptible and resistant varieties to *Al*Kewell82-GFP during early, mid and late phase. Inset in ILL6002 mid-infection phase, magnified image of enlarged hyphae localised within the leaf. Scale bars are 50 μm.

To investigate the interaction between *Al*Kewell82-GFP and different lentil genotypes, we performed a whole plant infection assay on the susceptible ILL6002 and resistant ILL7537 accessions (**Fig 5)**. We divided the infection cycle into three phases as early, mid and late stages. The early phase consists of the conidial adhesion, germination and entry into the host that occurs from 0–5 DPI. The mid-phase refers to the first sign of the necrotic lesion on the susceptible variety where colonization of the epidermal layer was observed by confocal and light microscopy; from 6–9 DPI. The late stage refers to extensive necrosis and sporulation on the susceptible variety, typically from 10–14 DPI.

During the early stage of infection, *Al*Kewell82-GFP germinated and formed hyphae on the surface of both ILL6002 and ILL7537. Penetration attempts on both lentil genotypes were evident as elevated white fluorescence signals around the various infection structures. These fluorescence signals were localised within the area of attempted penetration sites, which indicates that these were likely hypersensitive responses by the host plants. Nevertheless, no discernible difference in response to *Al*Kewell82-GFP from either of the lentil genotypes was observed (**Fig 5 early**). Hyphal growth continued on the surface of both lentil genotypes for the next three days and except for the localised HR, host cells remained intact and no visible signs of necrotic lesions were observed for either genotype.

**Fig 6:**
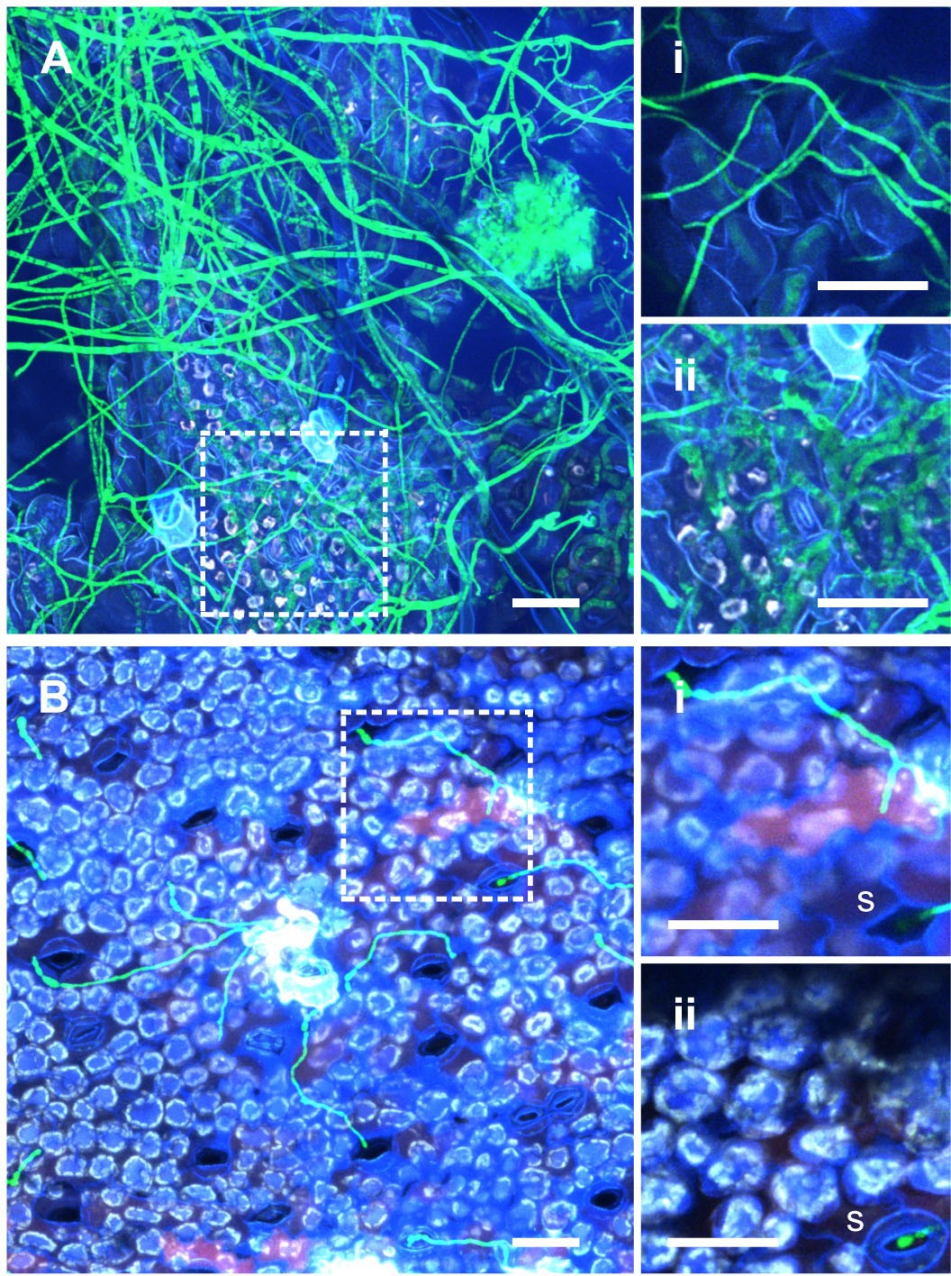
Observations of *Al*Kewell82-GFP at 7 DPI on ILL6002 (A) and ILL7537 (B) using confocal microscopy and optical sections at the leaf surface (i) and (ii) for each host genotype. Inset images are Z-projections of optical sections of the magnified areas indicated by the square on the main image (dotted line). For ILL6002, the leaf surface (A.i) and mesophyll (A.ii) sections were 11.2 μm and 12.8 μm in thickness and separated by 12.8 μm distance. For ILL7537, the leaf surface (B.i) and mesophyll (B.ii) sections were 4.8 μm and 9.6 μm in thickness, respectively, and separated by 4.0 μm distance. All scale bars are 50 μm.

At seven DPI, coincident with the first signs of macroscopic necrotic lesions in ILL6002, hyphal morphology around the lesion changed to a thicker, irregularly septated structure. In addition, increased hyphal growth and mycelial proliferation on the surface as well as colonization of the epidermal cells were observed. It appears that following the collapse of the epidermal layer, the pathogen continued to colonize the mesophyll, as we could clearly discern green fluorescent hyphae colonizing the plant cells below the sunken epidermal layer. On the other hand, long and thin filamentous hyphae were detected on the surface of ILL7537 (**Fig 5 mid**) with no apparent further differentiation in the hyphal structure and no colonization of epidermal or mesophyll tissues. At nine DPI, severe infection was clearly visible in the susceptible, ILL6002 leaves, manifested by the characteristic concentric black spots and sporulation on the leaf surfaces. Confocal images confirmed the extensive hyphal growth accompanied by formation of pycnidia (**Fig 5 late**). At this stage, ILL7537 remained free from necrotic lesions and *Al*Kewell82-GFP was almost completely absent from the surfaces of leaves. The differences in *A*. *lentis* colonization is further illustrated by viewing surface growth of hyphae and the colonization of internal leaf tissues by construction of separate Z-projections at the same leaf location for the two lentil accessions (**Fig 6A and 6B**). The distinct hyphal morphologies of surface, and epidermal and mesophyll-localised mycelium are clearly shown by selective optical sectioning. In **Fig 6B. i**, *A*. *lentis* has grown through a stoma on the resistant lentil, ILL7537, and neither a white fluorescence signal nor continuation of hyphal extension beyond the base of the stomatal cavity was observed (**Fig 6B. ii)**.

## Discussion

During plant-pathogen interaction, a well-orchestrated set of events occurs between the host and the pathogen that ultimately determines the progression and outcome of plant disease. The primary aim of this study was to characterise the disease progression of *A*. *lentis* during the full infection cycle from spore germination on the plant surface through to full colonization of the mesophyll and the production of asexual conidia, using a fluorescently-labelled *A*. *lentis* strain. In addition, differences in disease progression in resistant and susceptible lentil genotypes were explored. To help visualize and fully investigate the initiation and development of ascochyta disease in lentil, a fluorescent strain of *A*. *lentis* was produced using protocols developed for the genetic transformation of *Aspergillus awamori* [26]. This is the first report of the transformation of *A*. *lentis* and the use of an EGFP-expressing *A*. *lentis* strain to investigate the AB infection cycle in lentil. Successive stages of the infection process in whole plants were followed using CLSM up to 14 DPI, the stage at which macroscopic lesions indicate invasion and extensive fungal proliferation within infected lentil tissues. The use of fluorescently-labelled *A*. *lentis* enabled the investigation of the processes that occur inside the infected epidermal and mesophyll tissues that might otherwise be challenging to observe using histochemical dyes such as Trypan blue that stain fungal structures inside plant tissues with less efficiency and resolution than on the plant surface. Differences in the efficacy of the histochemical stain lactophenol cotton blue and the GFP-labelling approach have been demonstrated for *Colletotrichum truncatum* [34] where GFP shows higher resolution for labelled fungal cells in infected capsicum specimens than stained infected samples. In addition, CLSM enables imaging of invading mycelium through different optical planes, and image reconstructions across multiple optical sections to be produced. The results presented here illustrate the usefulness of the combination of a fluorescently labelled fungal isolate and CLSM for studies of lentil-*A*. *lentis* interactions.

ATMT has been widely adopted and proven to be an efficient tool for genetic transformation in filamentous fungi [35,36]. Several studies have reported the use of ATMT to transform fungal pathogens to express reporter proteins such as GFP and DsRed [23,37,38]. For the genetic transformation of *A*. *lentis* in this study, a new binary plasmid, pATMT-GdpGFP, was developed. USER^TM^ cloning technology facilitated the single step fusion of the respective components of the desired construct, that were derived from donor plasmids pEAQ-HT-DEST1 [25] and pGpdGFP [20]. The resulting plasmid contained the fungal GpdA and TrpC promoters that drive the expression of the *egfp* gene and the selectable hygromycin phosphotransferase gene (*hph*), respectively. The transformant, *Al*Kewell82-GFP, was able to express EGFP both *in vitro* and *in planta* (S2 Fig), which confirms that the GpdA promoter from *A*. *nidulans* is recognised by *Al*Kewell. As ATMT is known to insert transgenes randomly [39], the insertion site and number of T-DNA copies integrated into the genome of *Al*Kewell82-GFP were assessed by genomic sequencing. Approximately 3.5 tandem repeats of the whole binary plasmid inserted into the *Al*Kewell82-GFP genome, of which four full copies of T-DNA fragment remained intact (Fig 1). Tandem T-DNA insertion has been reported previously in the screening of *F*. *oxysporum* f. sp. *lycopersici* T-DNA mutants [39]. In addition, different patterns of T-DNA integration have been reported such as multiple T-DNA integration, inverted repeats, abortive T-DNA integration and the presence of non-T-DNA or binary vector for other fungal pathogens [20,23,39]. DNA sequencing of *Al*Kewell82-GFP showed a single insertion site within the intron of a gene annotated by InterProScan as a glycosyltransferase. The transformant remained stable after growth on agar plates without selection pressure and exhibited wild type growth characteristics (Fig 2). More importantly, there was no evidence that T-DNA integration had led to disruption of the normal capacity for the strain to infect lentil and to cause disease. Whole genome sequencing for characterizing transformants and assessing transgene copy number is more informative than the conventional Southern blotting method, with the ability to describe the exact location of insertion and the nature of the DNA inserted into the target organism. It is very likely that other *A*. *lentis* isolates would also be amenable to genetic manipulation using ATMT.

To gain insight into the basis of the full AB infection process, disease progression on susceptible and resistant lentil genotypes was monitored using CLSM, focusing on the development of infection structures, penetration and subsequent colonization of the host (Figs 3–6). Our research approach using GFP-labelled *A*. *lentis* follows the study of Sambasivam et al. (2017) [14] where early infection structures in the first 48 hours after *A*. *lentis* inoculation were characterized on detached lentil leaves. We observed that using the whole-plant infection assay, spore germination and development of infection structures on the leaf surface occurring over the first three days after inoculation exhibited no difference in germination rate between resistant and susceptible lentil genotypes (Fig 5 early). We suspect that the swollen hyphal structures at sites, indicated by associated fluorescence signals from adjacent host cells, are playing a role in penetration. However, these structures could possibly be hyphopodia that are more specialized towards nutrient uptake from the host rather than penetration [22]. Differences in plant cuticle properties between host species such as rice and maize, and in this case, lentil may have a bearing on the size and shape of penetration structures of plant pathogenic fungi and that the small hyphal swelling that we observe for *A*. *lentis* may be sufficient to breach the comparatively weak barrier of the lentil plant surface. Occasionally, direct penetration attempts of host cells were noted, similar to the penetration strategies documented for other necrotrophic filamentous fungi, such as *A*. *rabiei* [23] and *P*. *nodorum* (formerly named *Stagonospora nodorum*) [22]. It seems that the preferred mechanism of host entry is through the junction between the epidermal cells, either through direct germ tube penetration or formation of an appressorium. Stomatal penetration was also observed but there seems to be no preference for stomatal entry. This suggests that at least for this strain of *A*. *lentis*, stomatal invasion is a random process similar to *Zymoseptoria tritici*. [40,41], and in contrast to the observations reported by Roundhill et al. (1995) [13] in the infection of lentil cultivars, Laird and Invincible, where stomatal penetration of *Ascochyta fabae* f. sp. *lentis* (syn: *A*. *lentis*) was not observed.

Recognition by the host was evident during the early stage of infection where attempts by *A*. *lentis* to enter the host cells trigger a hypersensitive response (HR) and the release of reactive oxygen species (ROS). Sambasivam et al. (2017) [14] showed that the resistant genotypes elicited an earlier HR response as a defense mechanism against the invading *A*. *lentis* pathotypes using DAB staining. In our results, ROS was evident from brown DAB staining around both *Al*Kewell82-GFP and wild type *Al*Kewell on ILL6002 (Fig 5C–5D). Host cell death was confirmed in propidium iodide staining of ILL6002 cells attacked by *A*. *lentis*. In addition, the presence of white autofluorescence signals from epidermal cells at the site of invading *A*. *lentis* was a clear indicator of the host response by both the susceptible ILL6002 lentil and the resistant ILL7537 (Figs 3–4). It can be inferred that the white autofluorescence is a result of the phenolics and phenolic-conjugates released by the host as a defense response. Different phenolic compounds emit light at different wavelengths, and in this case, the white autofluorescence is a co-localization of light from the four detector channels and not from any one signal in particular. In the infection of barley by *Blumeria graminis* f. sp. *hordei* (*Bgh*) the host produces the defensive structures called papillae. These consist of the cell wall polysaccharides arabinoxylan and cellulose deposited at the site of fungal penetration of the epidermal cell wall [42]. Phenolic compounds accumulate at the site of penetration and the papilla forms at this site through polysaccharide deposition and cross-linking, and cell-wall strengthening by the incorporation of phenolics into the polysaccharide matrix [42]. We speculate that similar processes are occurring during pathogen attack of lentil by *A*. *lentis*. Chowdhury et al. [42] report that the level of autofluorescence attributed to phenolics at the site of penetration for *Bgh* in barley was not different for effective papillae that could ultimately prevent infection, and ineffective papillae that were unsuccessful in resisting the pathogen. Our results where penetration-site autofluorescence, which we speculatively ascribe to phenolics, was similar for both susceptible and resistant lentil accessions, were consistent with the findings for the proposed model for pathogen-associated papillae in barley [42]. Evidence of papilla formation as an early host defense response to *A*. *lentis* infection was reported by Khorramdelazad et al. [18]. In the transcriptome analysis of ILL6002 and ILL7537, the authors reported the upregulation of xyloglucan endotransglucosylase/hydrolase (XTH), and the differential regulation of laccase diphenol oxidase (PPOI) and Exocyst subunit 70A1 (*EXO70A1*), genes involved in cell wall restructuring and papilla formation. These genes were overexpressed in both lentil genotypes which suggests that a structural defense response is part of the host artillery to prevent pathogen spread [18]. Papilla formation has been reported to function as a barrier and may act as a first line of defense to the invading pathogen [42]. Our results show that changes in autofluorescence signals detected by CLSM at the site of attempts at penetration in resistant lentil and successful penetration in susceptible lentil genotypes, are highly sensitive indicators of plant defense responses towards *A*. *lentis* in histology studies of lentil-pathogen interactions. With minimal manipulation of infected leaf samples, the use of EGFP-labelled *A*. *lentis* and the fluorescence-based CLSM method allows both fungal cells and fluorescent plant responses to be observed concurrently.

During the early stage of infection, both susceptible and resistant lentil varieties remained asymptomatic where leaves appeared healthy and no macroscopic lesions were observed for the first 6 days. A long latent phase is also observed for the hemibiotrophic wheat pathogen *Z*. *tritici* [43]. The production of numerous proteases by *Z*. *tritici* during the proposed hemibiotrophic phase indicates that the pathogen may live on plant resources by metabolizing the apoplastic proteins and starch released from chloroplasts. In addition, host-adapted cell wall degrading enzymes are produced during early stages to avoid the basal wheat defense. The long latent phase observed in this study supports the idea that *A*. *lentis* is a hemibiotroph where the pathogen is asymptomatic for a prolonged duration after inoculation and then switches to the necrotrophic phase to colonize the host [13,15].

Colonization of the mesophyll of the susceptible lentil genotype coincides with the onset of macroscopic lesions, usually 6–8 days after inoculation. Microscopically, the onset of necrotrophic phase is characterized by the collapse of the leaf tissues (Figs 5–6). At this point, fungal hyphae invade the sub-epidermal layer and the mesophyll cells start to collapse, leading to visible signs of necrosis. Fungal morphology changes to enlarged and thicker structures with irregular septation and increased vacuolation. This is in contrast to colonization by the pathogen on the resistant lentil genotype, where surface fungal hyphae remained thin and did not form enlarged and complex subcellular structures. In addition, there was minimal proliferation on the leaf surface of the resistant lentil accession and further colonization of the mesophyll was not observed.

Our observations indicate that both resistant and susceptible lentil genotypes can recognise *A*. *lentis*, as evidenced by similar autofluorescence signals at penetration sites that are likely associated with phenolic compounds. Other defense responses are upregulated upon recognition of the pathogen by the host and these have been shown to differ in resistant and susceptible lentils. Transcriptome profiling during early stages of infection performed by Khorramdelazad et al. [18] on the same lentil genotypes used in this study, ILL6002 and ILL7537, infected with *A*. *lentis* revealed the differential expression of early plant defense response genes such as PR2, PR4 and PR10. Several other protein kinase receptors involved in pathogen recognition and early signalling, such as leucine-rich repeat (LRR) receptor-like kinase and calmodulin domain protein kinase (CDPK) were also differentially regulated. This is in agreement with the transcriptome analysis done by Sari et al. [17] on lentil varieties with non-allelic R genes, CDC Robin and 964a-46. In both studies, *A*. *lentis* was able to evade recognition by the susceptible host and continued to colonize the mesophyll. Degradation of the host cells provides nutrients to the invading pathogen that leads to the spreading hyphal growth during the asymptomatic phase followed by the transition to necrotrophy and the subsequent formation of pycnidia. Release of pycnidiospores serves as inoculum that enables infection of neighbouring plants and signals the start of the next infection cycle.

Detached leaf assays are often used to study early *in planta* growth stages of plant pathogenic fungi because they are convenient and often yield uniform results. However, Dadu et al. [16] observed a significant difference between detached leaf assays and whole plant inoculations for *A*. *lentis* in lentil. The authors observed that excision of leaves enhanced the susceptibility to *A*. *lentis* regardless of the resistance status of the lentil host genotype. Detached leaf assays for *Arabidopsis thaliana* similarly showed a distinct reaction by the hemibiotroph *Colletotrichum* spp [44] that was different to the plant response normally observed for whole plant infections. Excision of leaves may activate hormone defense-related signalling and may trigger leaf senescence [44]. Thus, the whole plant infection method reported here replicates the natural state of plant infection and interaction between pathogen and host.

Here we have presented new genetic transformation and microscopy methods that build upon previous work to further our understanding of ascochyta blight disease progression in lentils. The development of a transformation protocol for *A*. *lentis* opens the door to introducing other foreign genes and reporter tags to study molecular mechanisms underlying pathogenesis. This will also enable the study of virulence mechanisms using reverse genetics approaches by means of a targeted gene replacement or gene knock-out strategies to study the functions of particular genes. Furthermore, *Agrobacterium*-mediated transformation is a powerful technique to create a library of strains carrying random T-DNA insertions and mutations. Overall, the use of EGFP-expressing, and potentially other fluorescently labelled *A*. *lentis* strains, together with confocal microscopy will be valuable for studying interaction mechanisms among complex fungal communities in plant host systems. New strategies for observing plant-pathogen interactions will be useful in the search for resistant genotypes in lentil germplasm collections and in future breeding programs for evaluating the effectiveness of *R* gene-mediated resistance. Understanding processes and resistance mechanisms operating in lentil during its interaction with *A*. *lentis* is of utmost importance for the development of effective and durable ascochyta blight-resistant lentil cultivars.

## Supporting information

S1 Table List of primers. (DOCX)

S2 Table Summary of genome assembly statistics for Illumina sequencing of *Al*Kewell82-GFP. (DOCX)

S1 Fig Schematic diagram of the transformation vector (A) pATMT-GpdGFP amplified from pEAQ-HT-DEST1 and pGpdGFP and (B) the T-DNA region. Illustration was prepared using SnapGene Viewer (GSL Biotech; available at snapgene.com).
(EPS)

S2 Fig Expression of EGFP in *Al*Kewell82-GFP. (A) *Al*Kewell82-GFP grown on ½ PDA solid media; (B) *Al*Kewell82-GFP mycelia on ILL6002 at three DPI; (C) *Al*Kewell82-GFP pycnidia containing pycnidiospores on ILL6002 at 14 DPI.
(EPS)

S3 Fig Integration and coverage of pATMT-GpdGFP in *Al*Kewell82-GFP. (EPS)

S4 Fig Mycelial growth of *Al*Kewell and *Al*Kewell82-GFP on ½ PDA plates. (EPS)

S5 Fig Formation of pycnidia on ILL6002. (EPS)

S1 File Sequence of *Al*Kewell82-GFP transgene geneious format. (GENEIOUS)

S2 File Sequence of *Al*Kewell82-GFP transgene FASTA format–text file. (TXT)
