# Supplementary material for: Agrobacterium tumefaciens-mediated transformation and expression of GFP in Ascochyta lentis to characterize ascochyta blight disease progression in lentil
Source: PLoS One. 2019 Oct 24;14(10):e0223419. doi: 10.1371/journal.pone.0223419 (PMC6812748; doi:10.1371/journal.pone.0223419)
Supplement: S1 Table — (DOCX) [file pone.0223419.s001.docx]

S1 Table. Primer sequences used in this study

| **Name** | **Sequence (5’ –> 3’)** |
| --- | --- |
| JD278 | ACGGCACCUGTGCTTTACGGCACCTCG |
| JD287 | ATCTATGUCAGTACATTAAAAACGTCCG |
| JD292 | ACATAGAUTTAATTCTATTTGTGTTTGATCGAGACC |
| JD293 | AGGTGCCGUCAGAAGATGATATTGAAGGAGC |
| JD381 | TGGCAAAGCTCGTAGAAC |
| JD321 | AAGATCCGCCACAACATC |
| JD425 | AGAAAGCCCAGGATCCAT |
| JD427 | AAACAGCGTCGGATATCG |
