# Supplementary material for: Agrobacterium tumefaciens-mediated transformation and expression of GFP in Ascochyta lentis to characterize ascochyta blight disease progression in lentil
Source: PLoS One. 2019 Oct 24;14(10):e0223419. doi: 10.1371/journal.pone.0223419 (PMC6812748; doi:10.1371/journal.pone.0223419)
Supplement: S2 Table — (DOCX) [file pone.0223419.s002.docx]

S2 Table. Summary of genome assembly statistics for Illumina sequencing of *Al*Kewell82-GFP

| Total read length | 4.43 Gbp |
| --- | --- |
| Genome Assembly size | 41.4 Mbp |
| Coverage | 107x |
| Number of contigs | 563 |
| Largest contig | 1.05 Mbp |
| N50 (contigs) | 262,395 |
| L50 (contigs) | 46 |
